# Modulation of Arachidonic Acid Metabolism in the Rat Kidney by Sulforaphane: Implications for Regulation of Blood Pressure

**DOI:** 10.1155/2014/683508

**Published:** 2014-03-09

**Authors:** Fawzy Elbarbry, Anke Vermehren-Schmaedick, Agnieszka Balkowiec

**Affiliations:** ^1^School of Pharmacy, Pacific University Oregon, 222 SE 8th Avenue, Hillsboro, OR 97123, USA; ^2^Department of Integrative Biosciences, Oregon Health & Science University, Portland, OR 97239, USA; ^3^Department of Biomedical Engineering, Oregon Health & Science University, Portland, OR 97239, USA

## Abstract

*Background*. We investigated the effects of sulforaphane (SF), the main active isothiocyanate in cruciferous vegetables, on arachidonic acid (AA) metabolism in the kidney and its effect on arterial blood pressure, using spontaneously hypertensive rats (SHR) as models. *Methods*. Rats were treated for 8 weeks with either drinking water alone (control) or SF (20 or 40 mg/kg) added to drinking water. Mean arterial pressure (MAP) was measured at 7-day intervals throughout the study. At the end of treatment rats were euthanized, and kidneys were harvested to prepare microsomes and measure enzymes involved in regulation of vasoactive metabolites: CYP4A, the key enzyme in the formation of 20-hydroxyeicosatetraenoic acid, and the soluble epoxide hydrolase, which is responsible for the degradation of the vasodilator metabolites such as epoxyeicosatetraenoic acids. Effect of SF on kidney expression of CYP4A was investigated by immunoblotting. *Results*. We found that treatment with SF leads to significant reductions in both, the expression and activity of renal CYP4A isozymes, as well as the activity of soluble epoxide hydrolase (sEH). Consistent with these data, we have found that treatment with SF resisted the progressive rise in MAP in the developing SHR in a dose-dependent manner. *Conclusion*. This is the first demonstration that SF modulates the metabolism of AA by both P450 enzymes and sEH in SHR rats. This may represent a novel mechanism by which SF protects SHR rats against the progressive rise in blood pressure.

## 1. Background

**Figure 1:**
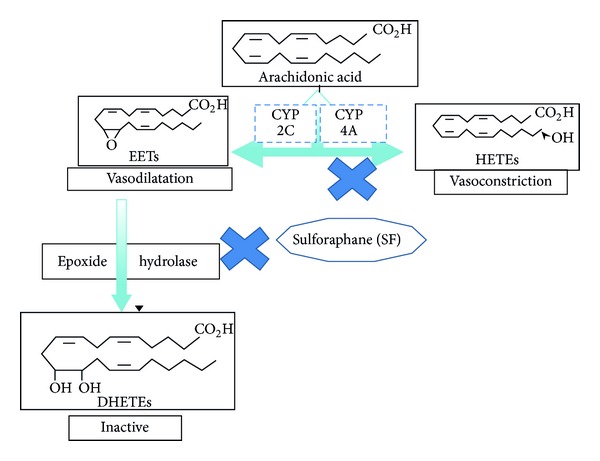
Metabolism of arachidonic acid by CYP enzymes and epoxide hydrolase. The sign “x” indicates the proposed sites for the antihypertensive effect of SF.

Hypertension affects about one in three adults in the United States and is a major predictor for coronary heart disease and stroke. Although there is still uncertainty about the pathophysiology of hypertension, many interrelated factors have been found to contribute to persistent blood pressure elevation [1]. Among the proposed factors are vascular resistance, oxidative stress, endothelial dysfunction, and salt sensitivity. Evidence from numerous studies indicate that arachidonic acid (AA) metabolites play an important role in the regulation of renal vascular tone, tubuloglomerular feedback, and sodium transport [2–4]. Therefore, we postulate that AA metabolites may participate in the pathogenesis of hypertension [2–4]. AA is metabolized by renal cytochrome P450 (CYP) enzymes to produce either hydroxyeicosatetraenoic acids (HETEs; particularly, 19- and 20-HETE) or epoxyeicosatetraenoic acids (EETs, Figure 1) [2, 3, 5, 6].

In the renal and peripheral vasculature, 20-HETE is a potent vasoconstrictor that enhances calcium influx through voltage-sensitive calcium channels [4, 7]. Moreover, the vasoconstriction response to agents such as angiotensin II and norepinephrine has been linked in several studies to the endogenous formation of 20-HETE in renal vascular smooth muscle and blockade of the K_ca_ channels [8, 9]. Specifically, infusion of inhibitors of the 20-HETE formation into the renal artery was found to attenuate the vasoconstrictor effect of these agents [7, 10]. Although 20-HETE has been shown to inhibit sodium reabsorption at the level of the renal tubules, it nevertheless promotes hypertension, primarily due to its effects, to increase vascular tone in the afferent arterioles and augmenting tubuloglomerular feedback responses [2, 3]. In the human kidney, two enzymes (CYP4A11 and CYP4F2) were found to be responsible for the production of 20-HETE [2]. In the rat, CYP4A1 was found to exhibit the highest catalytic activity for the formation of 20-HETE, followed by CYP4A2 and then CYP4A3 [11]. While CYP4A1 and CYP4A3 are preferentially expressed in the proximal tubules, CYP4A2 is highly expressed in the outer-medullary and thick ascending limb of the loop of Henle [12]. This site-specific expression may explain the preferential regulation and diverse actions of 20-HETE in the kidney.

The phase of rapid elevation of blood pressure in the spontaneously hypertensive rat (SHR) is associated with increased production of 20-HETE by CYP4A isoforms expressed in the renal tubules and blood vessels [13]. Conversely, reduction in the synthesis of 20-HETE prevents the development of hypertension in SHR [14]. Consequently, modulation of AA hydroxylase activity (i.e., 20-HETE-forming activity) could potentially have a significant impact on the development and progression of hypertension. Such modulation can occur in response to administration of drugs, herbal remedies, or even dietary supplements.

On the other hand, EETs, produced by the endothelium, are potent vasodilators [15]. Several studies have led to the proposal that EETs serve as the endothelium-derived hyperpolarizing factor (EDHF) through increasing the activity of a large-conductance, calcium-activated K^+^ channel (K_ca_) [15]. Deficiency in EETs, especially 5,6-EET, renders the rat liable to salt-induced elevations of blood pressure [16]. The human CYP2C is the main CYP epoxygenase gene family expressed in the human kidney, with CYP2C8, CYP2C9, CYP2C18, and CYP2C19 being the predominant isoforms responsible for the metabolism of arachidonic acid to biologically active EETs [17]. The three major CYP2C isoforms expressed in the rat kidney and responsible for most of the renal epoxygenase activity are CYP2C11, CYP2C23, and CYP2C24 [3]. The CYP2J family was also found to contribute to the formation of EETs in both human and rat kidneys [18]. High-salt diet increases the expression of CYP2C23 and formation of EETs [19]. Significantly, an increased conversion of EETs to inactive metabolites, dihydroxyeicosatrienoic acids (DiHETEs), by soluble epoxide hydrolase (sEH) has been reported in the SHR [3] (Figure 1). Consistent with the latter findings, treatment of SHRs with inhibitors of sEH lowers blood pressure [3, 20].

The idea of modulation of drug-metabolizing enzymes as a principal means by which dietary constituents affect the risk of disease was first outlined by Wattenberg [21] and was further elaborated by Prochaska and colleagues [22]. This idea was particularly applied to the natural phytochemical sulforaphane glucosinolate (SGS), which is present in the cabbage family of plants (kale and cabbage). SGS is metabolized to sulforaphane (SF), a potent inducer for phase II detoxification enzymes [23]. Young sprouts of cruciferous plants (e.g., broccoli) are extremely rich dietary sources of SGS [23]. Multiple human epidemiological studies, as well as animal experimentation, indicate that SGS consumption decreases the probability of developing hypertension, atherosclerosis, and cancers [23–26]. More recent research indicates that the cardioprotective and cancer chemopreventive effects of broccoli sprout consumption are primarily attributed to SGS being metabolized to SF [24, 27, 28]. The current study was undertaken to examine the antihypertensive effect of SF in a hypertension rat model and to investigate its effect on AA-metabolizing enzymes in the kidney.

## 2. Materials and Methods

### 2.1. Materials

Purified sulforaphane was obtained from LKT laboratories (St. Paul, MN). Arachidonic acid and 20-HETE were purchased from Cayman Chemical Company (Ann Arbor, MI). Sprague-Dawley rat kidney microsomes for* in vitro* studies were purchased from BD Biosciences (Woburn, MA). NADPH and all reagents used for microsomal preparation, determination of protein content, and enzyme assays were purchased from Sigma-Aldrich (ST. Louis, MO). CYP4A1/2/3 polyclonal antibody was purchased from LifeSpan Biosciences, Inc. (Seattle, WA). Acetonitrile and methanol were high pressure liquid chromatography (HPLC) grade and obtained from Fisher Scientific (Pittsburg, PA). All other chemicals used were analytical grade.

### 2.2. Animals

Male 14-week-old spontaneously hypertensive rats (SHR) were obtained from Charles River Laboratories (Wilmington, MA). All animals were maintained under controlled housing conditions of light (6 AM–6 PM) and temperature (22°C) and received standard laboratory chow and water* ad libitum*. All rats were allowed at least 2 weeks to become acclimated to the housing conditions and ensure steady and reliable blood pressure readings before use in experiments. All procedures were approved by the Institutional Animal Care and Use Committee of Pacific University and Oregon Health & Science University).

### 2.3. Sulforaphane (SF) Treatment

Animals were randomly divided into 3 groups of 5 animals each. Group one served as the control group, whereas groups two and three were provided with sulforaphane in the drinking water at concentrations of 20 mg/kg and 40 mg/kg, respectively, for 8 weeks. Preliminary stability studies using LC-MS indicated that SF is stable in water at room temperature for at least 5 days (data not shown). Fresh SF solutions were made from stock twice a week. The selection of SF doses was based on preliminary studies in our laboratory and previous studies [24, 26].

### 2.4. Mean Arterial Pressure (MAP) Measurements

Blood pressure was measured as previously described by Vermehren-Schmaedick et al. [29]. All blood pressure measurements were performed in conscious animals. For each SHR rat, blood pressure was measured once every week using a noninvasive method based on determining the tail blood volume with a volume-pressure recording sensor and an occlusion tail-cuff (CODA system; Kent Scientific, Torrington, CT). Our previous studies indicate that blood pressure values obtained with the noninvasive tail-cuff method are not significantly different from the values obtained in the same animals by direct measurements in the femoral artery [29]. Blood pressure was measured by multiple readings in individual rats, until an average of 15 stable measurements was obtained. Results are shown as the average of MAP values obtained from individual rats.

### 2.5. Tissue Collection

At the end of the study, rats were anesthetized with isoflurane; the abdominal cavities were opened, and the kidneys were rapidly removed and rinsed with ice-cold saline. Kidney tissues were then flash frozen in liquid nitrogen and stored at −80°C until use.

### 2.6. Preparation of Microsomes and Measurement of Protein Content

Kidney microsomes were prepared as described previously [30]. Briefly, kidney samples were homogenized in 2 mL ice-cold homogenization buffer (50 mM Tris buffer, 150 mM KCl, 0.1 mM dithiothreitol, 1 mM ethylenediaminetetraaceate (EDTA), 20% glycerol, and 0.1 mM phenylmethylsulfonylfluoride) using a Polytron homogenizer. The homogenate was centrifuged at 10000 ×g for 30 min at 4°C in a Sorvall MTX 150 Micro-Ultracentrifuge (Thermo Scientific, Asheville, NC). The supernatant was carefully transferred to clean ultracentrifuge tubes and centrifuged at 100 000 ×g for 30 min at 4°C. The supernatant (cytosol) was removed and transferred to labeled tubes and stored at −80°C until use. The pellet was washed in 2 mL of ice-cold 150 mM KCl and centrifuged again at 100 000 ×g for 30 min at 4°C. The pellet was resuspended in 2 mL of ice-cold 0.25 M sucrose solution and aliquots were stored in cryogenic microcentrifuge tubes (400 *μ*L) at −80°C until use. Microsomal protein concentrations were determined in triplicate by the method of Lowry et al. [31] using bovine serum albumin as a calibration standard. Absorbance was measured at 750 nm on a Synergy 2 microplate reader using Gen5 Software (BioTek, Winooski, VT).

### 2.7. Microsomal Arachidonic Acid (AA) Hydroxylation and Analysis of 20-HETE Formation

Oxidation of AA to its metabolite 20-HETE was determined in reaction mixtures (500 *μ*L) containing 100 mM phosphate buffer (pH 7.4), 40 *μ*M AA, 2 mM MgCl_2_, 1 mM NADPH, and rat kidney microsomes equivalent to 0.4 mg protein. Reactions were initiated with NADPH and were terminated after 15 minutes at 37°C with 1.0 M HCl and vigorous shaking.* In vitro* experiments using renal cortical microsomes from untreated Sprague-Dawley rats were similarly utilized to study the effect of SF on 20-HETE formation* in vitro*. In these experiments, microsomes were exposed to different SF concentrations (0.5, 12.5, and 50 *μ*M) in presence and absence of NADPH. 20-HETE formation rate in both* in vitro* and* in vivo* studies was measured as described previously [32]. Briefly, the reaction mixtures were extracted with ethyl acetate and the organic extracts were evaporated with nitrogen gas and the residues were reconstituted in the HPLC mobile phase. AA and 20-HETE were resolved on an Agilent Eclipse Plus C_18_ column (4.6 × 250 mm; Agilent Technologies, Santa Clara, CA) with UV detection at 200 nm. Initial mobile phase composition was 45% acetonitrile in water with 0.1% acetic acid. Linear gradient (0.5%/min) was utilized over 30 min, followed by a sudden increase of 20%/min to 100% acetonitrile. Flow rate was maintained at 0.3 mL/min and column temperature at 40°C.

### 2.8. Immunoquantification of CYP4A in Rat Kidney Microsomes

Rat kidney microsomes (10 ug per lane,) were incubated at 100°C for 5 minutes in Laemmli sample buffer (BioRad, Hercules, CA) and electrophoresed 1 hour constant voltage (150 V) through precast polyacrylamide gels (BioRad) with minor modifications [33]. Microsomal proteins were then transferred electrophoretically (30 min at 20 V) to PVFD membrane and incubated with CYP4A1/CYP4A2/CYP4A3 rabbit anti-rat polyclonal antibody, at a 1 : 1000 dilution (Lifespan Biosciences) overnight at 4°C. After washes with PBST, the secondary antibody (goat anti-rabbit conjugated to horseradish peroxidase, Thermo Scientific) was added at a 1 : 5000 dilution for 1 hour at room temperature. The immunoreactive proteins were detected via enhanced chemiluminescence and X-ray film imaging and the resultant signals were analyzed by densitometry (BioRad). The intensity of the band was normalized to GADPH signals, which was used as loading control.

### 2.9. Epoxide Hydrolase Activity (sEH)

Metabolism of Epoxy Fluor 7 (Cayman Chemical Co.) to a fluorescent metabolite was utilized to determine the sEH activity in kidney microsomes as described previously [34]. Briefly, reactions were carried out in mixtures (200 *μ*L) containing 25 mM bis-tris HCl, 1 mg/mL BSA, Epoxy Fluor 7, and rat kidney microsomes (equivalent to 10 *μ*g protein). The resulting solution was incubated at 37°C in a black 96-well flat bottom plate. The fluorescence of the Epoxy Fluor 7 metabolite was determined using an excitation wavelength of 330 nm and emission wavelength of 465 nm on a Synergy 2 microplate reader using Gen5 Software (BioTek).

### 2.10. Data Analysis

Data are reported as mean ± SD. Differences in 20-HETE formation rate, CYP activity, and level of protein expression and differences in MAP among groups were assessed by one-way analysis of variance (ANOVA) with Tukey's post hoc test for pair-wise multiple comparisons. Correlation analysis was also performed to investigate whether a change in blood pressure corresponds to a change in CYP4A or sEH activities. Statistical analysis was conducted using GraphPad Prism 5.0 (GraphPad Software Inc., San Diego, CA).

## 3. Results

Exposure of rats to different doses of sulforaphane (SF) had no obvious adverse effect and the gain in body weight was not significantly different in treatment groups compared to control animals (data not shown).

### 3.1. Effect of Sulforaphane Treatment on Mean Arterial Pressure (MAP)

**Figure 2:**
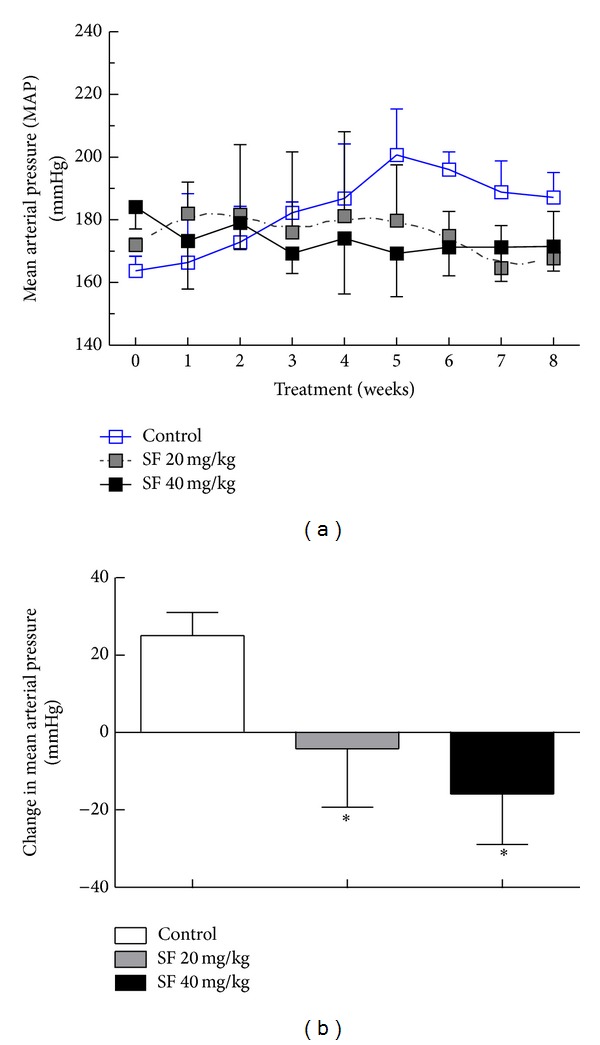
Effect of SF on MAP in SHR rats. Male 14-week-old SHR rats (*n* = 5/group) were exposed to SF in their drinking water at concentrations of 0, 20, and 40 mg/kg for 8 weeks. MAP was measured noninvasively once every week. Change in MAP is expressed as mean ± SD of 5 animals. (a) Changes in MAP in SHR over 8 weeks; (b) difference in MAP between the end and the beginning of the 8-week treatment, that is, (MAP at week 8)-(MAP at week 0). *Significant difference from control with *P* < 0.05.

To determine the effect of SF on blood pressure in SHR rats, we measured the MAP of all groups once a week.  Figure 2(a) shows the MAP in rats during baseline conditions and after exposure to low dose (20 mg/kg) or high dose (40 mg/kg) of SF. During the study, control rats showed a progressive rise in their MAP from 163 ± 13 to 187 ± 8-mm Hg after 8 weeks, an average increase of 25 ± 5.5 mm Hg. In contrast, exposure of SHR rats to even the low dose of SF prevented the expected progressive rise in MAP with a net decrease of 4.5 ± 15 mm Hg. Moreover, administration of SF in the high dose resulted in a more substantial decrease in MAP from 184 ± 7 at the beginning of the study to 171 ± 7.8 at the end of the 8-week treatment, with a net decrease of 14.9 ± 13 mmHg. The MAP in both low- and high-dose SF treatment groups showed a significant reduction when compared to the control group at the end of the 8-week treatment (Figure 2(b), *P* < 0.05) without a significant difference between the two SF treatment groups.

### 3.2. Effect of SF Treatment on Arachidonic Acid (AA) Metabolism

**Figure 3:**
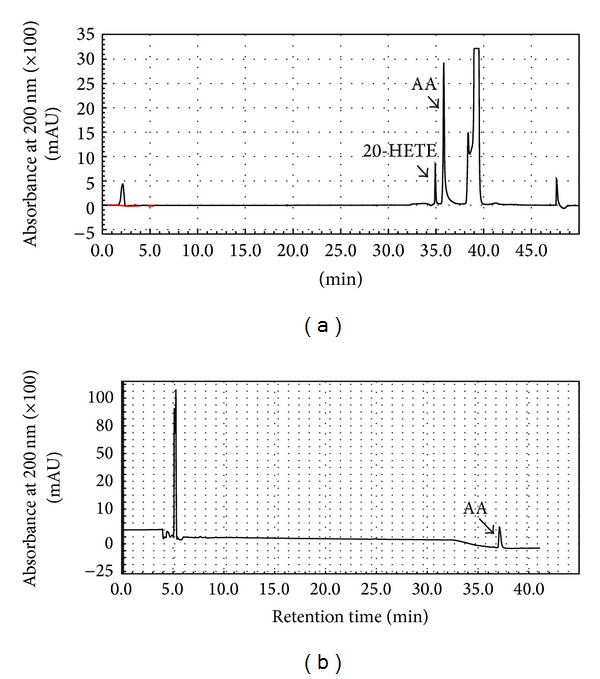
HPLC analysis of AA metabolism by rat kidney microsomes. (a) Representative chromatogram after incubation of AA with kidney microsomes from SHRs treated with 20 mg/kg SF in drinking water for 8 weeks. (b) Representative chromatogram produced after incubation of AA with untreated Sprague-Dawley rat kidney microsomes in the absence of NADPH. Additional details of the reactions and chromatographic conditions are described under Section 2.

**Figure 4:**
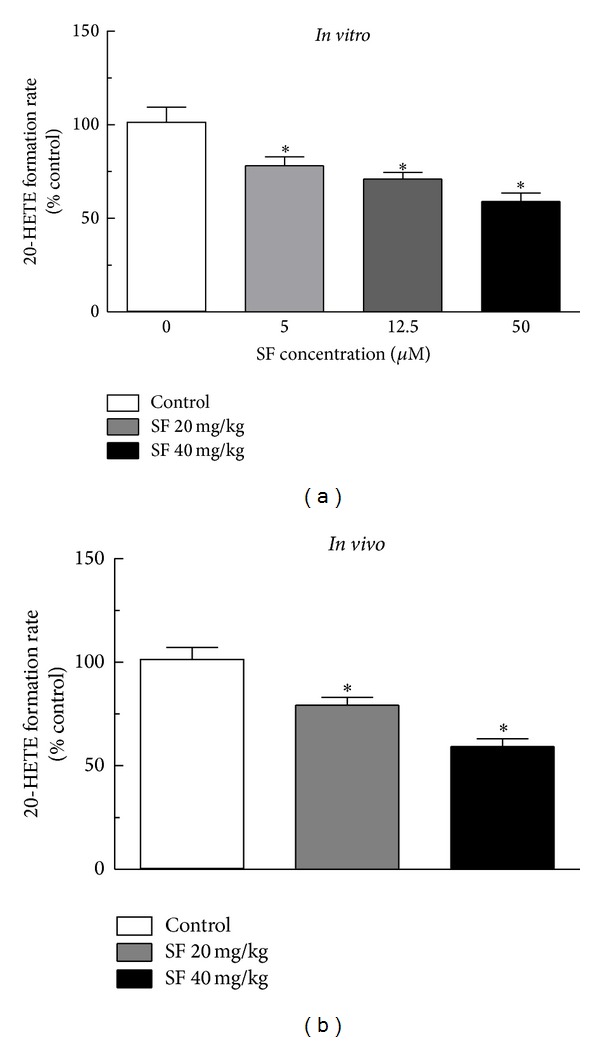
Inhibition of AA *ω*-hydroxylation activity by SF both* in vitro* and* in vivo*.* In vitro:* renal cortical microsomes from untreated Sprague-Dawley rats were incubated with various concentrations of SF in the presence of NADPH. SF-treated microsomes were then used to measure 20-HETE formation. Values, average of 5 replicates, are expressed as % of control (SF-free microsomes).* In vivo*: male SHR rats (*n* = 5/group) were administered 20 mg/kg or 40 mg/kg SF in their drinking water for 8 weeks and kidneys were harvested at the end of the study. The NADPH-dependent formation of 20-HETE was measured in renal cortical microsomes. Values are expressed as % of control (rats that received SF-free water). All groups were compared using one-way ANOVA followed by multiple comparisons. *Significant difference from control with *P* < 0.05.

Previous studies have shown that CYP4A is the predominant P450 isoform in the rat kidney responsible for catalyzing the *ω*-hydroxylation of (AA) to the potent vasoconstrictor metabolite, 20-HETE. To examine whether the observed reduction in blood pressure in SHR rats by SF could be associated with inhibition of CYP4A-mediated formation of 20-HETE, we first measured this reaction* in vitro* using renal cortical microsomes from untreated Sprague-Dawley rats. Microsomes were preincubated with or without SF (5, 12.5, and 50 *μ*M) in the presence of NADPH before adding AA (100 *μ*M) and other incubation components. An HPLC-UV method was utilized for the quantification of 20-HETE in the incubation mixture and measurement of 20-HETE formation rate as previously described [2, 32]. Under these chromatographic conditions, 20-HETE and AA were eluted with retention times of 35 and 37 min., respectively (Figure 3(a)). Additionally, and as shown in Figure 3(b), 20-HETE formation was NADPH-dependent. This may indicate that inhibition of 20-HETE formation by SF is mechanism-based as NADPH was required in the incubation mixture for inactivation of CYP enzymes. 20-HETE formation was inhibited by* in vitro* inactivation with SF in renal cortical microsomes in a concentration-dependent manner. At a SF concentration of 50 *μ*M, 20-HETE formation rate was reduced to 57–63% of control (Figure 4,* in vitro*).

The effect of the* in vivo* administration of SF on AA metabolism was measured in rat renal microsomes after 8 weeks of exposure to SF in the drinking water at concentrations of 20 mg/kg and 40 mg/kg. Similar to the observed* in vitro* results, AA *ω*-hydroxylation was inhibited significantly by both low-dose and high-dose SF compared to control rats, with no significant difference between the two SF treatment groups (Figure 4,* in vivo*).

### 3.3. Effect of SF Treatment on CYP4A Protein Levels

**Figure 5:**
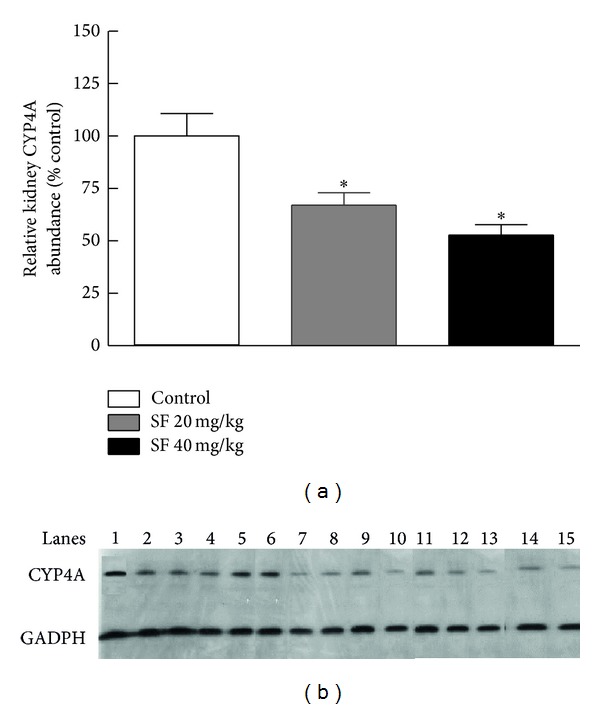
Effect of SF on kidney expression of CYP4A. (a) Renal CYP4A protein levels in rats (*n* = 5/group) treated for 8 weeks with SF at 20 mg/kg and 40 mg/kg expressed as the percentage of control levels in untreated rats. The immunoreactive proteins were detected via enhanced chemiluminescence and X-ray film imaging and the resultant signals were analyzed by densitometry. The intensity of the band was normalized to a loading control (GADPH signals); then data were expressed as percent of control. (b) The original specimen analyzed by densitometry (control: lanes 1–5, low SF dose: lanes 6–10, and high SF dose: lanes 11–15). *Significant difference from control with *P* < 0.05.

To examine the effect of SF on the expression level of CYP4A protein, western blotting of renal microsomes was performed using a polyclonal antibody against rat CYP4A1/2/3. As shown in Figure 5, inhibition of AA *ω*-hydroxylation was associated with a loss of CYP4A immunoreactive protein. Similar to the observeddose-dependent inhibition of AA hydroxylase activity, SF also caused a significant inhibition of CYP4A protein expression compared to the control group, but no significant difference was observed between the two SF treatment groups (Figure 5).

### 3.4. Effect of SF Treatment on Soluble Epoxide Hydrolase (sEH) Activity

**Figure 6:**
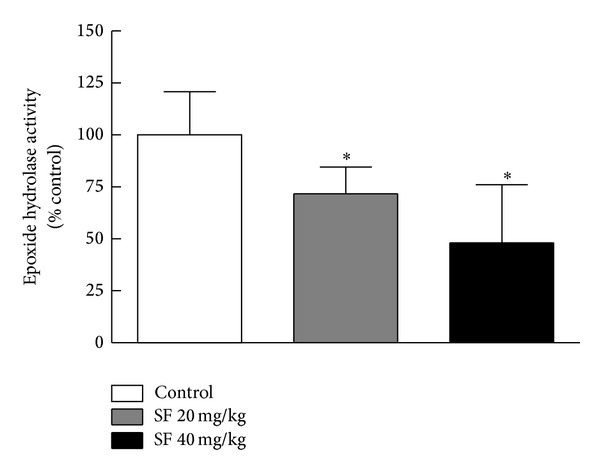
Effect of SF on activity of kidney sEH. Mean epoxide hydrolase (sEH) activity in rats (*n* = 5/group) treated for 8 weeks with SF at 20 mg/kg and 40 mg/kg expressed as the percentage of control fluorescence in rats that received SF-free water. Rat kidney microsomes (equivalent to 10 *μ*g protein) were incubated with Epoxy Fluor 7, and the fluorescence of the Epoxy Fluor 7 metabolite was determined. Data are presented as mean ± SD. *Significant difference with *P* < 0.05.

sEH plays a major role in the metabolic conversion of the cardioprotective eicosanoids to inactive metabolites. To further study the effect of SF treatment on sEH activity, kidney microsomes from control rats or rats exposed to different concentrations of SF were utilized as explained briefly in Section 2 [34]. The results shown in Figure 6 indicate that sEH activity was significantly inhibited by 30% and 51% following SF treatment with doses of 20 mg/kg and 40 mg/kg, respectively. No significant difference was observed between the two treatment groups.

**Figure 7:**
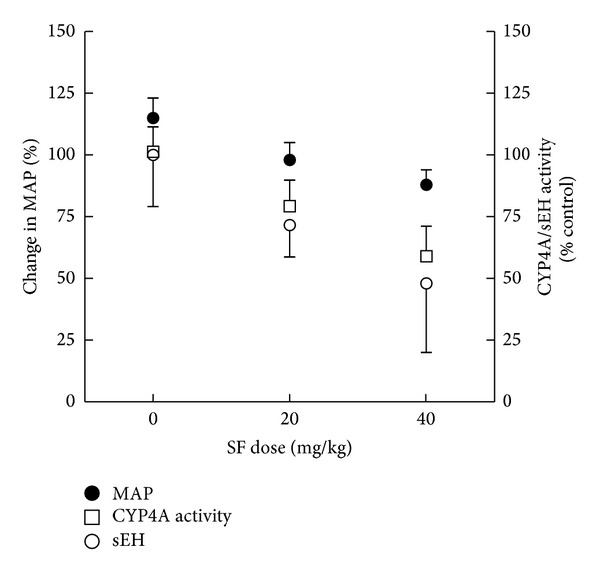
Correlation of changes in MAP, CYP activity, and sEH activity as a function of SF dose. Percent changes in MAP (closed circles) in correlation to CYP4A activity (open squares) or sEH activity (open circles) as a function of SF dose. MAP is presented as % change from baseline (week 0). Both CYP4A and sEH activities are presented as a % of control activity. While treatment rats received SF in their drinking water in doses of 20 mg/kg or 40 mg/kg for 8 weeks, control rats did not receive SF.

Since establishing a cause-effect relationship between the observed effect of SF on MAP, CYP4A activity, and sEH activity was not possible with our study, we investigated the correlation between these variables. When SF-dependent changes in MAP were compared to changes in 20-HETE formation rate as the measures of CYP4A activity, a good correlation was observed (Pearson *r*; −0.992 and *P*; 0.041). While 20 mg/kg and 40 mg/kg SF doses reduced MAP by approximately 8 and 12%, 20-HETE formation rate was reduced by 21 and 41%, respectively (Figure 7). Similarly, changes in MAP reasonably correlate (Pearson *r*; −0.9954 and *P*; 0.046) with changes in sEH activity in a SF dose-dependent manner. Namely, compared to 8 and 12% reduction in MAP, sEH activity was reduced by 30 and 51% following administration of 20 and 40 mg/kg SF, respectively (Figure 7).

## 4. Discussion

This study was undertaken to investigate possible mechanisms that contribute to the beneficial effect of sulforaphane (SF) on blood pressure in hypertensive rats. The results show for the first time that treatment of spontaneously hypertensive rats (SHR) with SF significantly prevents the progressive rise in blood pressure, reduces the expression and activity of renal CYP4A isozymes responsible for 20-HETE formation, and reduces the activity of soluble epoxide hydrolase (sEH) responsible for the degradation of the vasodilator metabolites EETs.

SF is a naturally occurring compound that exhibits cytoprotective, antioxidant, and anti-inflammatory vasoprotective properties [35]. Most studies examining the mechanism of action of sulforaphane glucosinolate (SGS) proposed that preventing and/or inhibiting the development of cardiovascular diseases is related to the ability of SF to reduce oxidative stress and associated inflammation and, thus, ameliorate diseases that become more common with aging, such as hypertension [26, 36]. For example, the consumption of broccoli sprouts rich in SGS decreases oxidative stress in spontaneously hypertensive-stroke prone rats (SHRsp), lowering their blood pressure and decreasing inflammation in all tissues examined, including heart, arteries, kidney, and central nervous system [24, 26]. Furthermore, when pregnant SHRsp were fed with this source of SGS, their adult offspring had lower blood pressure and less inflamed organs than the parental generation [27]. Whether this antihypertensive effect of SGS is due to antioxidant effect is not yet established. Additionally, if the antihypertensive effects of SF result exclusively from its antioxidant properties, then other antioxidants should also show similar antihypertensive properties. However, results of three randomized clinical trials testing the effects of the antioxidant vitamin E on hypertension have proven disappointing [37–39]. Similarly, a nonsignificant reduction in blood pressure was reported in vitamin D-treated patients [40]. Accordingly, it is conceivable that SF reduces blood pressure by more than one mechanism.

This study is the first to investigate the effect of SF on modulation of AA-metabolizing enzymes in the kidney, as a potential mechanism for its antihypertensive property. Even though AA and its metabolites are present in numerous organs, including the liver, lungs, and brain, the kidney plays a central role in arterial blood pressure regulation through both local and hormonal mechanisms. AA metabolites, such as 20-HETE, are known to have a strong vasoconstrictive effect on the renal afferent arteriole that, in turn, is likely to trigger the powerful renin-angiotensin-aldosterone (RAA) cascade, one of primary targets of antihypertensive therapy. The fact that 20-HETE also has a direct inhibitory effect on sodium reabsorption, which is opposite to the RAA-mediated sodium retention, is likely to explain the less pronounced changes in blood pressure during a global downregulation of 20-HETE formation, as in the current study.

To avoid inducing any stress-related effect on blood pressure and also to simulate the commonly used route of SF administration, we introduced SF to rats in their drinking water rather than oral gavage. Our stability studies using LC-MS indicate that SF is stable in water at room temperature for at least 5 days. Also, several studies have indicated that SF is bioavailable in both human and animal models [41, 42]. Selection of SF doses was designed to reflect the normal daily servings of broccoli, that is, clinically relevant doses. Broccoli naturally contains glucoraphanin, not SF. The conversion to the more active SF takes place either by the action of the enzyme myrosinase, also present in fresh broccoli, or in the body during the digestion process. However, this conversion is highly variable. Assuming 100% conversion and considering the molecular weights of glucoraphanin (436 g/mole) and sulforaphane (177 g/mole), one serving of broccoli (10 oz.), which contains approximately 30 mg of glucoraphanin, will release approximately 12 mg of SF. Administration of 20 mg/kg and 40 mg/kg of SF to SHR rats with a body weight range (400–600 g) is expected to provide 8–24 mg of SF daily.

Notably, the observed attenuation of the expected rise in blood pressure by SF after an 8-week period was comparable to the results achieved with commonly used antihypertensive medication in the same rat model. For example, telmisartan (5 mg for 8 weeks), indapamide (1 mg/kg for 8 weeks), hydrochlorothiazide (20 mg/kg for 8 weeks), and metoprolol (100 mg/kg) with hydralazine (50 mg/kg; for 10 weeks) resulted in an average MAP reduction of 25, 17, 15, and 22 mmHg, respectively [43]. Such reduction in blood pressure was associated with a similar pattern of inhibition in the formation rate of 20-HETE in kidney microsomes. It is well established that 20-HETE induces hypertension through its potent vasoconstriction in the renal and peripheral vasculature and by promoting sodium retention [3]. Considerable evidence has now accumulated suggesting that development of hypertension in the SHR strain is, at least in part, attributed to the elevated production of 20-HETE in the kidney and mesenteric arteries [14]. Our data are consistent with this hypothesis and with several findings that inhibitors of 20-HETE synthesis lower blood pressure by 10–15 mmHg in SHR [14, 44]. In attempts to identify the cytochrome P450 (CYP) isoforms responsible for the renal metabolism of AA and formation of 20-HETE using selective inhibitors and antisense oligonucleotides, it has been identified that CYP4A1 exhibits the highest catalytic activity for the formation of 20-HETE in the rat kidney, followed by CYP4A2 and CYP4A3 [7, 19]. Our immunoblotting data using specific antibody against CYP4A1/2/3 indicate that SF lowers the expression of these isoforms in a manner similar to that involved in reducing MAP and 20-HETE formation.

It has also been established that EET metabolites of AA produced by the endothelium are potent vasodilators and natriuretic agents and are proposed as endothelium-derived hyperpolarizing factors [15, 45]. Degradation of these metabolites by sEH led to more research interest towards inhibiting this enzyme as an avenue to increase EETs levels and prevent progression of hypertension [45]. In addition to elevated renal production of 20-HETE, SHRs also exhibit an increased expression of renal sEH compared to their normotensive WKY control rats [13, 18]. Specific inhibitors to sEH enzyme lowered blood pressure in SHR, but not in WKY rats [13, 18, 46]. Such findings may indicate that sEH contributes to the initiation of mechanisms that eventually result in high blood pressure in SHR. Our data indicate for the first time that both low and high SF doses inhibit the activity of sEH in the kidney of SHR, and this inhibition was parallel to the observed reduction in blood pressure.

## 5. Conclusion

Our results have shown that even the lower SF dose prevented the progressive rise in MAP, while the higher SF dose significantly reduced MAP in hypertensive rats. We demonstrated for the first time that the lowering of blood pressure was accompanied by modulation of AA metabolism through inhibiting both, the formation of 20-HETE and activity of sEH. Those results contribute novel knowledge to the antihypertensive properties of SF.

Future studies will be undertaken to examine whether an earlier exposure to SF can prevent the development of hypertension and whether SF can reverse the established hypertension in older SHR rats. Additionally, we will examine the effect of specific inhibitors or inducers for the 20-HETE formation pathway on the observed antihypertensive effect of SF in SHR rats.
